# Autoethnography of a Novice Teacher’s Assessment Literacy in Elementary Physical Education

**DOI:** 10.1080/17482631.2021.1882066

**Published:** 2021-02-02

**Authors:** Youngjoon Kim, Okseon Lee

**Affiliations:** a Seoul Misung Elementary School, Seoul, Korea; b Department of Physical Education, Seoul National University, Seoul, Korea

**Keywords:** Physical education assessment, elementary physical education, assessment literacy, autoethnography, novice teacher

## Abstract

**Purpose**: The purpose of this study was to examine how a novice teacher (researcher) has developed his assessment literacy in elementary physical education (PE), and thereby investigating what cultural, micropolitical and sociological factors have impacted on his enactment of assessment literacy.

**Method**: Adopting autoethnography, this study investigated   theresearcher as a subject and an object of research in the pursuit of extending the personal to the social. The 4 years of narrative data collected from the researcher’s reflective journals, self-recalling, and artefacts on PE assessment were analysed using structural narrative analysis and reiterative process.

**Findings and discussion**: The findings revealed that thenovice teacher developed his assessment literacy in a chronological order: (a) assessment illiterate, (b) assessment literate, and (c) assessment aliterate. The cultural, micropolitical and sociological factors that have impacted on the teachers’ enactment of assessment literacy were discussed: (a) rampant complacency in elementary teaching culture: a bad judge or a good bystander?, (b) uniform culture of grade-level teams, and (c) distorted PE professionalism focusing on “hows”, not “whys”.

**Conclusion**: The novice teacher’s enactment of assessment literacy in elementary PE was not only related to himself but also to the school culture, grade level team, and PE professionalism where he belonged.

## Introduction

Students do not always learn what they are taught in education. The assessment process is, thus, indispensable for effective education to ascertain what students have actually learned by eliciting and interpreting evidence (Black & Wiliam, 2018). Recently, as a counteraction to an overemphasis on the summative assessment and accountability (Hay, 2006), a rising discourse of assessment for learning (AfL) has gained reputation for a dominant school-wide assessment trend for several decades (Deluca et al., 2012). Posing three questions of “where is the learner going?—where is the learner right now?—how does the learner get there?” (Wiliam, 2011, p. 12), AfL has focused on the learners who were once marginalized in traditional assessments. Accordingly, growing demands of national curriculums and educational policies for teachers to integrate AfL into practice (Birenbaum et al., 2015; Korean Ministry of Education, 2015) have taken teachers to the role of AfL facilitators.

Despite the national and policy promotions of AfL discourses, teachers are still facing difficulties with PE assessments in their teaching practices (Yoo, 2005). Under the umbrella of AfL philosophy, the assessment, in alignment with curriculum and pedagogy, is supposed to serve as one of the three-message systems in education (Bernstein, 1990). However, PE assessments are still recognized as an afterthought and remain disconnected from learning (Hay & Penney, 2013) by teachers who are already accustomed to Tyler’s (1949) curriculum model asserting that the assessment is simply located at the last stage of education (Moon et al., 2016). Literature has reported that AfL has not been integrated into educational practices thoroughly because of teachers’ misconceptions of AfL philosophy, theory and practice (E. Hargreaves, 2005), perceived misalignment between system accountability priorities and teachers’ assessment practices (Gardner, 2006), and the lack of effective models on professional development for assessments (C. Lee & Wiliam, 2005). More specifically, in PE contexts, the issues such as teachers’ indifference to assessments, lack of knowledge about assessments, and marginalized assessments compared with pedagogy and curriculum have consistently been identified as critical challenges in implementing PE assessments. (Annerstedt & Larsson, 2010; Hay & Penney, 2013; Matanin & Tennehill, 1994).

Thus, the importance of teacher variables regarding applying assessment knowledge and skills in a wide range of classroom settings as part of their teaching practices (Harlen & Gardner, 2010) cannot be overemphasized. To succeed in integrating AfL into their practices, teachers should be assessment-literate. The concept of assessment literacy in physical education proposed by Hay and Penney (2013) provides not only fundamental and technical elements in practising AfL, but also an alternate perspective on how the assessment in itself is socio-culturally constructed. PE teachers should be aware of the fact that the assessment cannot be inevitably neutral in the valuing process of determining “what to assess and what not to assess” (Hay & Penney, 2013; Park, 2017). The assessment literacy is the prerequisite for teachers that should be attained properly through pre- and in-service teacher education, yet some studies indicate inconsistent or weak assessment literacy development in initial teacher education (ITE) programmes (Maclellan, 2004; Volante & Fazio, 2007). Furthermore, most novice teachers report being inadequately prepared and feeling challenged when asked to design assessment tasks, ensure equity for all learners, and mark fairly and communicate assessment results (Koh, 2014; Smith et al., 2013).

However, the assessment literacy should not be merely considered as individual teacher’s ability. According to Willis et al. (2013), assessment literacy and illiteracy are not only about competence but also about the capacity to “become” assessment literate in particular settings or situations. Situation and contexts determine appropriate use of assessment, and consequently the same teacher can be assessment literate in one situation and assessment illiterate in another (Leirhaug et al., 2016). Thus, enacting assessment literacy is not only teacher’s competence which an individual “have” but as a capacity that teacher can actually “do” in various circumstances of where they belong, what cultures they are situated in, and who they work with. Despite the importance of the situations and contexts in enacting assessment literacy, most studies have merely focused on whether or not the desirable assessment literacy has appeared in an individual teacher (DinanThompson & Penney, 2015; Leirhaug et al., 2016; Macken et al., 2020) since the Hay and Penney’s (2013) first theoretical publication on assessment literacy in physical education. The limitation of these studies is that they only focus on the teachers’ individual competence demonstrated in their assessment practices through the assessment literacy framework, while overlooking sociological, cultural, relational and political factors impacting teachers’ assessment literacy. Furthermore, elementary teachers who are assigned multiple subjects have experienced relatively more difficulties in PE assessments than middle and high school PE teachers (Park, 2017), yet the studies dealing with elementary teachers’ assessment literacy is extremely scarce in number.

Thus, there needs to be an academic attention to the novice elementary teachers who implement PE assessments in their teaching practices as well as the cultural, micropolitical, and sociological factors in enacting teachers’ assessment literacy. To do so, this study adopted autoethnography as a research methodology because it enables the researchers to investigate deeply into their own experience and attendant emotions that may not be obtained if they were being interviewed by someone else (Ngunjiri et al., 2010). Because the novice teachers are heavily subject to their teaching culture with the experienced senior teachers (Rossi et al., 2015), this vulnerable positionality tends to make themselves hide and assume their lived experiences and emotionality when interviewed by others. Moreover, “to write individual experience is, at the same time, to write social experience” (Mykhalovskiy, 1996, p. 141). Since autoethnography is a qualitative research method using self-narratives to make personal occurrences become cultural things by seeking to understand individual stories in social contexts (Mayan, 2009; Reed-Danahay, 1997), this study attempts to produce the researcher’s personal stories of PE assessments as well as investigate sociological, micropolitical, and hegemonic factors impacting the enactment of assessment literacy in elementary PE by challenging “what counts as knowledge” (Morimoto, 2008, p. 31).

### Teacher’s assessment literacy in elementary physical education

In schools, assessment occupies a notable amount of teaching and learning time. Stiggins (1999) argued that typical teachers spent between 30% and 50% of their professional time for assessment activities. Therefore, teachers need to be assessment literate to fulfil the complicated demands of using assessment as a means to affirm, extend and account for student’s learning (Edwards, 2017) as well as to maximize the learning potential of assessment (DeLuca & Klinger, 2010). The assessment literacy is not a completely new concept in education. Since the first introduction of assessment literacy proposed by Stiggins (1991), this notion has been constantly refined and developed by many scholars. It is defined as “an individual’s understandings of the fundamental assessment concepts and procedures deemed likely to influence educational decisions” (Popham, 2011, p. 267).

In physical education, Hay and Penney’s (2013) definition of assessment literacy is widely accepted as a solid groundwork for developing assessment literacy discourses among PE scholars. The assessment literacy refers to as not only having technical qualities for assessment activities but also critically recognizing the sociocultural nature inherent in assessment practice which can never be neutral in power relations between teachers and students because of the valuing process of selecting what to assess and what not to assess, how to assess and deal with assessment results. Thus, the assessment literacy in PE suggested by Hay and Penney (2013) advocated that both teachers and students should be assessment-literate by possessing the capacity to enact with the technical procedures of PE assessment and having critical awareness of sociocultural influence in and on PE assessment that latent marginalization and negative consequences in the choices made by teachers.

Therefore, four inter-related elements of assessment literacy in PE presented as follows: assessment comprehension—focusing on knowledge and understanding of assessment expectations and conditions of efficacy; assessment application—focusing on the conduct of assessment in terms of either teaching, implementation or student engagement; assessment interpretation—focusing on making sense of and acting on the information that is collected through assessment practices, including traversing and negotiating the social relations of assessment; critical engagement with assessment—focusing on awareness of the impact or consequences of assessment and challenging the “naturalness” of assessment practices, performances and outcomes. (Hay & Penney, 2013, p. 73). These elements are relevant to both teachers and students, but this study is mainly dealt with teacher’s assessment literacy only.

Despite such significance of teachers’ assessment literacy in PE contexts, the academic interests of assessment literacy in elementary PE have been limited in number. Some studies investigated individual elementary teachers’ PE assessment practices (DinanThompson & Penney, 2015; Park, 2017), other research explored pre-service elementary teachers’ assessment literacy demonstrated during school placements while taking assessment training seminars (Macken et al., 2020). However, through the theoretical lens of the assessment literacy framework, these studies are likely to misattribute the assessment literacy merely as an individual’s competence or personal trait without considerations of cultural, micropolitical, and sociological factors influencing teacher’s enactment of assessment literacy.

It is partially true that assessment literacy is seen as a competence that individual teachers are expected to read and interpret in assessment practices. But, given that the interpretation and reading process are likely to be heavily influenced by both individual and collective beliefs about and value orientations regarding the subject in the geographical regions and cultures where they work (Curtner-Smith et al., 2018; Oh et al., 2013; Zhu et al., 2011), assessment literacy and illiteracy are not only about competence but also about the capacity to “become” assessment literate (illiterate) in particular settings or situations (Willis et al., 2013). Situation and contexts determine appropriate use of assessment and consequently, the same teacher can be assessment literate in one situation and assessment illiterate in another (Leirhaug et al., 2016). Thus, enacting assessment literacy is not only teacher’s competence which an individual “have” but as a capacity that teacher can actually “do” in various circumstances of where they belong, what cultures they are situated in, and who they work with. This study tried to illuminate on a novice elementary teacher’s 4 years of assessment practices and explore the cultural, micropolitical, and sociological factors in impacting on the enactment with assessment literacy through autoethnography.

### Researcher’s backgrounds as a novice elementary teacher in South Korea

It’s been 4 years since I (researcher) taught children in elementary school in Seoul, Korea. At first, I bore an ideal dream of being a professional elementary PE teacher. Moreover, I was quite confident that I was fully equipped with academic knowledge and theory from university. But it has been turned out to be useless in the real contexts since every single pedagogical moment that I encountered in my PE classes was always something new and unfamiliar to me. “Reality shock” (H. Lawson, 1983) was the only word that can most concisely explain myself at that time.

Time never solved the problem. The more time went by, the more I found myself I was a clumsy PE teacher compared with other senior teachers. To solve my problem, I started graduate study in sport pedagogy to become a professional PE teacher. I was provided the best qualities of learning associated with curriculum, pedagogy, and assessment in PE. During this period, by reflecting on my PE practices based on the up-to-date theory, my understandings of curriculum, teaching skills, and managing PE classes have been much more upgraded than before. However, one thing that I have never improved a bit was my PE assessment practices. I could not keep up with the latest academic discourses as well as national policies on assessment.

In Korea contexts, the national policies and educational discourses have constantly requested teachers to improve the traditional assessment. In 2009, the 7th revised national curriculum declared the enhancement of formative assessment as a counteraction to the summative and outcome-oriented assessment in the past (Korean Ministry of Education, 2009). Recently, the 2015 revised national curriculum proposed the process-oriented assessment in the pursuit of assessment for student’s learning (Korean Ministry of Education, 2015). This viewed students as active learners and teachers as learning facilitators who design assessment properly and monitor students’ learning process. Even though some might think that this sounded perfect for making assessment a learning process, it rings hollow to me.

During my 4 years of teaching career, PE assessment at school was the most unchanged area that fewer corrections were made than curriculums and pedagogy in PE classes. My and my colleagues’ assessment practices were mainly conducted for measuring and collecting students’ physical information to assign scores for accountability. This rule of thumb from my experiences is also corroborated by multiple research (Buns, 2015; Kirk, 2010; O. Lee, 2018). Of course, I have tried myself to become an AfL facilitator, but failed in the end. It was not an easy task for a novice teacher alone to innovate assessment practices which had been implemented dominantly among grade level teachers, because most school cultures are very conservative in nature (Zeichner & Tabachnik, 1981). This settling culture of grade-level teachers was likely to maintain the status-quo, which is no room for a novice teacher to introduce new assessment philosophy and even ask them to change their PE assessment practices.

Thus, the cultural, micropolitical, and sociological factors in novice teacher’s enactment of elementary PE assessment practices have to be re-illuminated for deeply understanding how the novice teacher’s positionality affects assessment literacy. Literature on PE teachers’ socialization suggests that the induction stage as teachers’ first few years of employment in the profession (Fessler & Christensen, 1992) is a critical period of transition that teacher experienced “self-doubt” and “uncertainty” (Woods et al., 2017). H. A. Lawson (1989) also highlighted that beginning educators must navigate the cultural norms within schools which are related to assumed societal expectations. These sociological factors cause novice teacher to have internal conflict (Richards et al., 2014) and lead to washout of skills and belief (Blankenship & Coleman, 2009). In addition, teachers in this period adjust to the teaching styles of their colleagues despite strong views formed during pre-service education (H. A. Lawson, 1989; Smyth, 1995) and using strategies “going with the flow” and “not rocking the boat” to avoid conflict with their experienced senior teachers. (Rossi et al., 2015). Given these facts, this study illuminated a novice elementary teacher (researcher) who struggled with enacting assessment literacy in PE.

### Purpose of the study

The purpose of this study was to examine how a novice teacher (researcher) has developed his assessment literacy in elementary physical education during his 4 years of teaching, and thereby investigating the cultural, micropolitical and sociological factors impacting on the novice teacher’s enactment of assessment literacy. Specific research questions that guided this study were: (a) How has a novice teacher (researcher) developed his assessment literacy in elementary physical education during his 4 years of teaching? (b) What cultural, micropolitical and sociological factors have impacted on the novice teacher’s enactment of assessment literacy in elementary physical education?

## Methods

### Autoethnography

In qualitative research traditions, autoethnography has a methodological value in using personal experiences to illustrate facets of cultural experiences. It reveals characteristics of a culture for the purpose of helping insiders (cultural members) and outsiders (cultural strangers) better understand the culture (Ellis et al., 2011, p. 275). It also aims to extend very personal stories from an author in order to develop a sociological understanding surrounding him/her (Sparkes, 2000). Rooted in ethnographical methodology, it uses personal autobiographies as raw materials and derives understandings of societies and cultures by interpreting the cultural relationship between the self and the culture where the self is situated (Chang, 2007).

Although autoethnography achieves the cultural understanding underlying autobiographical experiences by undergoing similar qualitative research processes which entails data collection, analysis, interpretation and writing (Chang, 2016b), it is distinguished from other qualitative research methods such as narrative inquiry and life history research. Narrative inquiry collects various forms of narratives (verbal, writings, visual data) from participants to gain insight into the complexity of human life (Trahar, 2009). The difference is that autoethnography explores narratives from the self, but narrative inquiry only focuses on retelling stories from other participants (Park et al., 2010). Life history research focuses on an individual’s life so strongly that the cultural aspects that has affected one’s life are not brought to the foreground.

According to Ellis and Bochner (2000), “auto” means focusing on the researcher’s autobiographical data, “ethno” means the main focus is on the culture, and “graphy” means the application of anthropological ethnography as a research methodology. Therefore, autoethnography is a written work about the sociological, political and cultural understandings of the self that are interrelated with others in a culture by reflecting one’s subjective lived experiences (S. H. Jones, 2005). Moreover, it is a form of self-study, which is historically derived from concepts and notions of reflective practice (Brown, 2011). It can also attempt to subvert dominant discourse (Muncey, 2010), criticizing the culture in operation.

Even so, autoethnographic studies in PE contexts have been limited in number, despite PE scholars’ constant promotions of autoethnography for teachers (Casey et al., 2018; McCree, 2019; Sparkes, 2000, 2002). Brooks and DinanThompson’s study (2015) is the most representative work written in autoethnography from physical education contexts. As a primary PE specialist in Australian school, the author’s autoethnographic accounts identify a sense of placelessness in his position in a performance-based culture as narrowing his enactment of democratic professionalism. It begins with his personal story but ends in a cultural story detailing the author’s deep reflections and greater cultural interpretations.

Therefore, this study was designed to utilize autoethnography to explore the phenomenon of elementary PE assessment in detail by investigating a novice teacher’s autobiographical descriptions. As a novice teacher, the author of this study reflects on his own PE assessment practices in an elementary school setting and seeks to discover cultural, political and sociological factors in his enactment of assessment literacy.

### Data collection

#### Self-reflective journal

The data were collected from the researcher’s self-reflective journals, daily classroom logs, and the fieldnotes that contained his reflexive writings from September 2016 to August 2020 during the 4 years of teaching. The self-reflective journal is not only a vehicle for revealing the self but also an interrogation of the intersections between self and society, the particular and the general, the personal and the political (Berry & Clair, 2011). The researcher started writing process in September 2016 and continued through August 2020. The self-reflective journal is the researcher’s record of memories and insights into what was experienced in relation to PE assessments. The daily classroom logs are the researcher’s thoughts and feelings and the students’ responses on the PE assessment classes. The fieldnotes were his own reflexive writings when he engaged in his Master’s thesis regarding elementary PE assessments. He wrote irregularly during this period of time. Consequently, he made 143 journal entries total. Most were free written in blank notebooks and the researcher’s reaction to the certain experiences of PE assessment.

#### Self-recalling

In addition, this study facilitated the practice of self-recalling (Chang, 2016b) to collect formal curricular meetings and informal conversations with grade-level teams, phone calls with colleagues, and self-talk related to PE assessments because those data can only be accessed and acquired by the self. For the most part, the data collected from official meetings, private conversations, self-talks were written in the researcher’s writings but some of data were not kept in written language because the incidents that the researcher encountered were so trivial that they just passed by in a flash. The self-recalling process might raise credibility and objectivity issues by a few social scientists because memories can be distorted arbitrarily and constructed selectively in the recalling process. However, other qualitative research also heavily relies on the memories from the researcher and the participants (Park et al., 2010). Therefore, this study adopted the self-recalling process as an alternative data sources in chronological order, while capturing the critical personal and social events (Chang, 2008).

#### Artefacts

Finally, cultural artefacts were collected to inspire critical events or thoughts on elementary PE assessments such as national curriculum documents, yearly assessment plans, process-oriented assessment guide books from the Office of Education, and computer messages related to PE assessments in the workplace.

### Data analysis

The autobiographical data from September 2016 to August 2020 were analysed in two ways. First, we formulated the storyline or plot of the researcher’s narratives on PE assessment based on the Labovian structural narrative analysis that the typical structure of narratives includes an abstract, orientation, complicating actions, evaluation, resolution, and coda (Riessman, 2008). Each element denotes: (a) abstract: a summary of the story and its points, (b) orientation: providing a context such as place, time, and character, (c) complicating action: an event that causes a problem, (d) evaluation: evaluative comments on events, justification of its telling or the meaning the teller gives to an event, (e) resolution: consequence of the story or the conflict, (f) coda: bridging the narrator and listener back to the present. The whole paper in this study has one storyline followed by the Labovian model of structural narrative. The abstract and orientation (a, b) were proposed in the introduction, theoretical framework, and researcher’s backgrounds section of this paper. The complicating action, evaluation, and resolution (c, d, e), which is the most important part in the study, were thematized as assessment-literate, -illiterate, -aliterate in the conclusion section while they were presented in a chronological order. The coda (f) was presented in the discussion and conclusion section.

Second, to extend the personal to the social through the autoethnography, we followed the reiterative process (Chang, 2016a; Ellis et al., 2011; Pheko, 2018). It includes: (a) self-observation and examination of realities and experiences of the phenomenon; (b) collection and verification of data (c) reviewing the research literature to compare my experiences with others’ experiences, as well as to understand the meaning of events and the content being analysed; (d) re-analysing and interpreting data to decipher personal meanings of events, behaviours and thoughts; and (e) writing the autoethnography. By following the repetitive procedure of comparison to others’ experiences, we tried to keep wary eyes on that the researcher’s narrative was not the only case.

### Establishing trustworthiness

Similar to other qualitative studies, ensuring validity and reliability are hard things to achieve. However, the issues of validity and reliability should be highly considered in light of the fact that autoethnography uses self-narratives as main data sources in studies. Thus, this study followed Richardson’s (2000) and Duncan’s (2004) criteria, ensuring validity and reliability. Firstly, not to indulge the initial author’s self-pity and egocentric bias, triangulation with a corresponding author and a graduate student was conducted. Secondly, to strengthen the contextuality, the researcher as both the subject and the object in this study, provided his positionality and the backgrounds in as a detailed manner as possible. Thirdly, by collecting data from multiple sources during 4 years of teaching as well as conducting a peer-review process with two colleagues in my elementary school, the study developed into a more reliable and credible one. Lastly, the researcher described the purpose and procedures of the study to the school principal as well as the colleagues who appeared in my narratives, asked for their consents before study, and shared the initial interpretation of the manuscript with them in order to abide by three ethical principles of respect for persons, beneficence, and justice in doing autoethnography (Adams et al., 2015). Every name appearing in my narratives is a pseudonym, and the consent from the school was also obtained.

## Findings

According to the Labovian model of structural narrative analysis (Riessman, 2008), the findings in this study were categorized by how a novice teacher developed his assessment literacy in practising PE assessments in a chronological order: Assessment-illiterate; -literate; -aliterate.

### Assessment illiterate (2016-2017)

In 2016, I was newly appointed as a public elementary PE teacher, a position which I very much wanted. Still, teaching in elementary school was a more complicated and nuanced activity than I previously expected. Educational theories and national curriculums that I mechanically memorized to enter this profession were useless when applied to school settings. I was desperate to thrive in my new position and wanted to do so just like other teachers. In order to overcome the “reality shock” (H. Lawson, 1983) as a newcomer, I was forced to grasp at straws, asking senior teachers’ help and choosing to follow their PE assessment practices blindly.

After finishing all my classes, I looked at the curriculum document in the empty classroom.

“Assessments should involve three balanced domains of psychomotor, cognitive, and affective domains beyond the traditional assessment practices focusing on the measurement of motor skills.”

I repeated it several times, something I had already done tons of times before becoming an elementary teacher. *But, what’s the point of this? It has nothing to do with my upcoming volleyball assessment class. How do I put all the domains together in my volleyball assessment?* I decided to drop by Mr. Cheon’s classroom and ask for help. Fortunately, he responded very kindly.

“Mr. Kim, I’ll be frank with you. In elementary school, we just evaluate kids on criterion-referenced assessments.” Mr. Cheon explained. “Don’t be too hard on yourself. I’ll suggest the two most effective ways to assess elementary PE that I strongly recommend: The first way is to let students repeat the desirable movements and practice them over and over to assess students’ final performances, a process which is easily measurable. The second way is to assign ‘good’ grades to every student regardless of their outcomes, so you can avoid potential objectivity issues which some parents might complain about. ‘Good’ grades would never be a problem, but ‘needs improvement’, which is the lowest grade, can cause some issues from students and parents. So, what role would you take? A bad judge or a good bystander? Think of it just as labor that both students and teachers are compelled to do. So, if you really want to know how to assess the affective domains in PE, my answer would be that you should become a good bystander, giving them ‘good’ grades blindly. You won’t get in trouble with assigning good grades, as my teaching career will show you. Plus, as elementary teachers, we can’t help finding the simplest and easiest ways to assess each subject because we don’t have enough time to design each assessment activity for over 10 subjects.” (Self-reflective journal)

More often than not, Mr. Cheon, who has 20-year teaching career, helped me out of various difficulties that I encountered at work as a new teacher. But, although his advice on PE assessments was technically helpful, I kept wondering why there was a stark gap between what I learned from university and the de-facto PE assessment practices in school contexts. What I learnt from university is that assessment should pursue the students’ learning. However, in reality, teacher’s assessment only cares about the accountability by grading. The two methods of elementary PE assessment that he recommended did not have any concerns about students’ learning or the educational purpose of assessment activities. Rather, his way of viewing PE was as not a learning activity, but a compulsory duty to which teachers and students must adhere. In addition, as elementary schools adopted criterion referenced assessments, he advised me to become either a judge or a good bystander in implementing PE assessments.

Although I had inner conflicts in response to his suggestions, I was not competent enough to plan and design an assessment aligned with curriculum and pedagogy. Because of my lack of capacities to plan and design assessments during this period of my career, there was no choice for me but to copy their practices.

(Pop-up message on my computer screen at work) “Our yearly plan for the PE assessment will go over the same as last year’s. It is not difficult at all for you to implement it into your classroom. So, please check out the attached document and let me know if it needs to be changed.” (Artifact: Computer message)

One day at work, I received a message from the grade head teacher through the computer messenger which is commonly used for teachers to communicate with each other. I thought we were supposed to have the 6th grade level team meeting for constructing a yearly plan for the PE assessment, but it did not work out this way. The decision-making procedure of planning the PE assessment was casually devised through the computer messenger without the teachers’ inputs or thoughts. Likewise, most elementary teachers at my school considered the PE assessment to be merely a daily and monotonous practice which is taken for granted, thus, for them it did not have to be something brilliant. They just needed a ready-made plan that could be used for the very next day’s PE class, so they could assign grades in order to exhibit a sense of accountability.

Ever since that experience, I started to ask myself about assessment practices and observed the senior teachers’ assessment practices at the playground through the windows of my classroom. They implemented assessments as directed by head teachers’ guidelines written in the attached document that the grade head teacher had previously sent. Rather than putting their efforts in preparing and implementing their own plans, they just managed to assess what they were asked to do in an easy manner without using or developing their own philosophies on PE assessments. By borrowing the assessment tools and standards from the previous year or by using pre-made ones obtained from online teachers’ communities or websites, they only used teaching methods to which they were already accustomed.

### Assessment literate (2018)

I named this period of time “assessment literate” for several reasons; I gradually gained some forms of literacy and professionalism related to PE, including the development of unique assessments. Firstly, I started studying sport pedagogy in graduate school while obtaining my Master’s degree in order to enhance my PE practices. Secondly, the Ministry of Education in Korea promoted the process-oriented assessment as an educational policy derived from the philosophical concepts of assessments for learning (AfL). Like it or not, around me, there was both an advanced academic environment and a reforming wind of change regarding assessments. By taking classes on PE assessments in graduate school and attending several seminars on process-oriented assessments provided by the government, I came to rethink the fact that PE assessments should target students’ learning and rather got to know the practical ways of planning and designing my own assessments. Little did I know that my journey towards enacting my own assessment literacies had obstacles waiting in front of it.

(At the grade level team meeting while sitting in a group) “To meet the increasing demand of the reformation of traditional educational assessments, we must also figure out a way to reform our own PE assessment this semester. Mr. Kim, now that you are studying PE in graduate school, you must have more knowledge on this matter. Any ideas?”

For the first time, I, a novice teacher, was the object of another teacher’s attention in the grade level team meeting setting. (After some deep breaths) “Um, first of all, I think we have to embrace the concept of AfL. Beyond its traditional purpose of measuring students’ movements and ranking them, PE assessments need to aim at helping students’ learning. The demand of integrating process-oriented assessments, which are promoted by our government, into our practices is underpinned by this concept.”

“I’ve heard about it too,” Mrs. Park said. “To me, process-oriented assessments sound too philosophical and unrealistic. For better or worse, we are obliged to assign grades for accountability. This fact will never change. Moreover, practical methods of learning must consider desirable outcomes in advance. Identifying and judging whether desirable outcomes have emerged or not is one of the most essential purposes of assessments. Traditional assessments have played such an identifying role. I don’t see any reason to change it.”

The grade head teacher then said, “I agree with Mrs. Park’s idea. Even though the new guideline for a process-oriented assessment was distributed by the Ministry of Education, it’s merely a collection of exemplars that are not fit for all. It is not too late to wait till the process-oriented assessment is well-established and settled. Practically, we don’t have enough time to think of each and every assessment in more than the 10 subjects we teach. What we need is an assessment method that we can use right now, in its right place. Since some members of our grade level team are not willing to change the PE assessment, then we will maintain our status-quo for this semester.”

I wanted to argue further but didn’t because I knew I was a novice at best who was too young in the teaching profession to dare change them. (Self-reflective journal)

I ended up failing to persuade the teachers in my grade level team to change our traditional assessment practices. The chance of innovating on the past assessments went up in smoke in the end because the suggestion I made was not accepted unanimously within my grade level team. What I wanted to do was to make little changes to the PE assessment collaboratively and see how they would affect the students’ learning. But, most of experienced teachers were too focused on the “hows” while making little of the “whys”. They instead opted for the most effective and objective tool for them to use right away in their next PE class while neglecting why such assessment method should be practised in the first place.

Despite the grade level team’s disapproval, I decided to change my assessment practices by myself. Once I proved that I could pave the way for enacting assessment literacy for students’ learning in PE classes, they would give me credit and thus participate in reforming their own assessment practices in the end. Thus, unofficially, I began integrating AfL concepts into my PE assessment practices while officially following the traditional assessments as agreed to in the grade level team meeting. Instead of using physical activities tests, I designed badminton PE assessments to enhance students’ learning. Included in this was a writing assignment assigned after the viewing of a badminton game, the reading of badminton books, peer-assessments, authentic-assessments of playing rallies, and video-taping of high-clear strokes with self-reflection. Moreover, I allowed my students to participate in every procedure of the PE assessment. Rather than being told how and what to be assessed, the students had the opportunity to communicate and negotiate with me during the entire process of the PE assessment. They enjoyed the PE assessment more than before and even became motivated by it. The more I demonstrated assessment literacy, the more students became assessment-literate, reconsidering themselves as the owners of both their own learning and the assessment.

### Assessment-aliterate (2019-2020)

Unfortunately, the good times did not last long. Other teachers in my grade level team seemed unhappy about what I had done alone, even if it was still deemed “unofficial.” Although not all, some teachers disliked the fact that a new teacher’s conspicuous practices were not in agreement with what had been previously discussed. In their eyes, I was the outlier who broke the code of conduct of shared and well-established PE assessments. I discovered such an atmosphere when having a conversation with Mrs. Jeon in the hallway one day.

“Mr. Kim, by the way, my students keep nagging me these days, saying they want to have fun activities in PE class,” Mrs. Jeon huffed. “They are comparing my class to your class. What on the earth did you do to them?”

“Ah … it’s nothing,” I said. “I just adopted what I learned in graduate school, but I did so additionally, apart from our shared PE assessments which were discussed in the grade level team meeting. In altering the PE assessment to meet their learning needs, I tried my best to make students the owners of their learning so they could be a part of the whole PE assessment process.”

“Additionally?” she questioned. “You adopted a new assessment into your class by yourself? Anyway, I’ve been having difficulties settling down my students in PE class … they keep making comparisons to your class.”

“I’m very sorry to make trouble, ma’am. I promise I’ll be careful.” (Self-recalling)

On one hand, after I noticed that other teachers had uncomfortable feelings about my practices, I started walking on eggshells around them. But, on the other hand, I felt greatly disappointed about the fact that a novice teacher could never innovate on the traditional, deep-rooted customs of elementary PE assessments. Regardless of how much educative effort I put in reforming the traditional PE assessment, I, a novice teacher, could not stand out from the grade level team. This is because, in elementary school, the assessments need to be practised equally as a team to balance the quality of practices among teachers within the grade level team.

Ever since that interaction, I became a lethargic teacher. Rather than teaching and assessing enthusiastically, I was as invisible as air in school, teaching and assessing just as other teachers do. Actually, I was being aliterate in my assessments. Aliteracy refers to the choice not to practice literacy skills in linguistic contexts (Agee, 2005). Vanderbilt (1999, p. 81) elaborated on this concept in detail: “Aliteracy has been defined as the ability to read without the desire to do so.” From the moment I was discouraged from restructuring the outmoded culture of PE assessment practices, I began demonstrating assessment-aliteracy. To be safe in this school as well as in the teaching profession, I had to hold my tongue and position myself as assessment illiterate.

I don’t need to stand out here and they won’t allow me to. If I really want to reform a culture of PE assessment, then I have to grow old enough to become an experienced teacher who has a strong will to embrace a good philosophy or method for improving outmoded traditional practices. I’d rather be just as still as a stone until then. (Self-talk)

Although I was knowledgeable about what PE assessments were and how they could be integrated into practice, I was not willing to do it. Purposefully, I didn’t read any new documents and guidelines on assessments supplied by the Office of Education. Just as senior teachers were reluctant to innovate on their own assessment practices in PE, I was gradually becoming similar to them. All I was concerned about was teaching and assessing just enough not to get any complaints from the principal, colleagues, students, and parents.

(During a PE assessment, in class) “Now, Subin, go ahead.” Subin comes out in front of me and performs some cheerleading. As soon as she finishes, I assign her a grade based on the scoring rubric constructed in the grade level team meeting. “Okay then, now you can play over there. Make sure you do not interrupt the next assessment. Next, Minjoo!” At this point, I had really deviated so far from the educational rhetoric’s ‘assessment for learning’. Now, I only thought about the report card. If there was no complaint, then I was good to go as far as I was concerned. (Self-reflective journal)

In a linguistic context, aliteracy is caused by the digital era which made young people gather information from sources such as the Internet and television because these sources are much more accessible, efficient, and easier to read (Agee, 2005). Likewise, my assessment aliteracy was also heavily affected by the culture of my grade level team in the pursuit of handy and familiar practices. At first, I refused it. But, once I got used to it, I was overwhelmed by the comfortable and easy assessments with no pedagogical considerations. Thus, the rhetoric of assessment for learning that I once pursued faded away, little by little.

## Discussion

### Rampant complacency in elementary teaching culture: A bad judge or a good bystander?

The purpose of elementary PE is to maximize students’ potential to help them feel safe as they enter a movement culture and ultimately participate in it continuously (Griggs, 2015). However, elementary PE assessment in South Korea has been falling behind in its purpose for elementary PE. The findings clearly mirror the “double edged sword” characteristic of elementary PE assessments (DinanThompson & Penney, 2018). On one hand, some teachers practised PE assessments by measuring and recording students’ movement based on the prescribed standards (Griggs, 2010; Kirk, 2010) to make it objective. On the other hand, other teachers implemented low-stakes assessments (DinanThompson & Penney, 2015), regardless of the PE curriculum, because of parents’ and students’ low expectations for PE (Han & Cho, 2016). Since South Koreans have long been under the Confucian tradition which puts emphasis on academic achievements, the academic achievement-oriented school culture (O. Lee & Choi, 2015) has prevailed in school contexts. In this era of “educational fever” (Kim et al., 2005), it is too natural that accountability in PE, not considered as an academic subject, is very low in the elementary school which has no impact on entering college.

Moreover, this ironic phenomenon of a “double edged sword” in elementary PE assessment is accelerated in its absurdity by the peace-at-any-price teaching culture. Findings showed that, in PE assessments, teachers intentionally assumed their role of either a bad judge to avoid receiving any complaints from students and parents for objectivity issues (Jung, 2014) or a good bystander to be comfortable in PE classes by giving “good” grades to all regardless of their learning. In this complacency teaching culture, teachers recognize the assessment practice merely as routinized labour of their workplace, taking advantage of the blind spot of criterion-referenced assessment, such as giving “good” grades to all, and going along with the social climate of the marginalization of PE in the era of academic fever. The students’ learning through PE assessments is not primary but secondary or periphery for teachers. Therefore, rampant complacency in elementary teaching cultures leads to depriving students’ learning opportunities from PE assessments as well as deskilling teachers’ assessment practices altogether (A. Hargreaves, 2001). This teaching culture was one of the significant factors holding back a novice elementary teacher from innovating and challenging the prevailed conservative PE assessment practices.

### Uniform culture of the grade-level team

The findings suggested that the grade level team to which a novice teacher belonged had a crucial impact on the shaping his assessment literacy in practising elementary PE assessments. In elementary school contexts in South Korea, the grade level team is a unique teaching culture organized in the beginning of the academic year and dismissed at the end of the year. Most elementary schools are administered by the same grade level team consisting of generalist teachers (Y. Lee & Cho, 2014), which is representative of elementary school culture (Jang & Lee, 2014). Grade level team meetings normally were held every day after school hours to utilize and maximize the collective intelligence in handling school issues, such as running annual grade-based curriculums and sharing educational knowledge and daily practices. Acknowledging the culture and propensity of fellow teachers, including senior and colleague teachers, powerfully socializes novice teachers who just entered the teaching profession (Choi, 2014). This peer socialization of the grade level team made the researcher internalize the values and behaviours of accountability and maintain a teaching profession based on it.

However, unlike the proper function of this grade level team, as findings showed, it negatively affected a novice teacher’s assessment practices. For a novice teacher, a grade-level team was representative of conservative school culture (Prior & Curtner-Smith, 2020) and uniform mechanisms for controlling what to permit and not to permit regarding best assessment practices. As finding illustrated, the researcher gradually became aliterate following ineffective assessment practices of senior teachers’ and even their modes of school life blindly because he wanted to be safe in the culture of the grade level team. Furthermore, this uniform culture worked for balancing the educational qualities of each class within a grade-level team in order not to receive any complaints from parents. Under the umbrella of grade-level team, the diversity of educational practices by each teacher was not guaranteed. A novice teacher’s educational trial to reform the traditional PE assessment practices was not accepted favourably by fellow teachers because he could not satisfy all the teachers in the grade level team. If every member consented to it, then it would be a “good to go” for every class, otherwise a “no go” for all. Instead of enacting assessment literacy for students’ learning in his class alone, a novice teacher strategically chose to position himself in this team by being assessment aliterate as well as holding his tongue to maintain cordial relations (Tinning & Siedentop, 1985) with senior teachers as a way to protect himself in this culture of the grade level team.

### Distorted PE professionalism focusing on “hows”, not “whys”

Unlike secondary schools administered by subject-specialist teachers, most elementary schools are run by generalist teachers who are responsible for teaching all subjects. Although elementary teachers are required to possess capacities and expertise in teaching every subject (Jang & Lee, 2014), many classroom teachers do not possess the skills or knowledge needed to deliver adequate PE lessons (Fletcher et al., 2013; Morgan & Bourke, 2008). The factors barring elementary teachers from building their PE professionalism were identified that they were lacking teacher training in the scope of PE (DeCorby et al., 2005; Morgan & Bourke, 2005) and overall confidence in their teaching abilities (Xiang et al., 2002). Given these facts, the findings illustrated elementary teachers’ realities, conflicting interests between their status-quo and the desired professionalism when implementing PE assessments. They borrowed assessment tools handed down from the past or from other teachers (Ahn et al., 2007) rather than reflecting on their own PE practices. Moreover, they obtained the PE assessment materials from the online and offline teacher’s communities and even mobile messengers as an “ambulance” for supporting PE (O. Lee et al., 2018, p. 11). Although it is easily accessible and adoptable to the next PE class, it could not extend the enhancement of these teachers’ pedagogical content knowledge (O. Lee et al., 2018).

Although, admittedly, we live in a pedagogized society (Bernstein, 1996) where the Internet, social media, or any kind of sources can be learning pools for teachers’ professionalism, the elementary teachers’ pursuit of PE expertise focusing on “hows” is not as good as teaching and assessing PE poorly. Because they only consumed PE assessment materials that someone already made, teachers’ practices lacked their own pedagogical insights on the rationales behind the assessment. D. Lee (2011) argued that we are situated in a teaching culture immersed in the method of “hows” and “how well” only, not asking “what” and “why” things should be taught. Furthermore, this overemphasis on “hows” in teaching accelerates the deskilling of teachers (Stremmel, 2002) and reproduces teachers’ images as technique-consumers and deliverers of prescribed curriculums. Thus, PE professionalism in elementary school is distorted and threatened by teachers’ culture that only focuses on “hows”, not “whys”.

## Conclusion

The aim of study was to examine how a novice teacher (researcher) has developed his assessment literacy in elementary physical education during his 4 years of teaching, and thereby investigating what cultural, micropolitical and sociological factors have impacted on the teacher’s enactment of assessment literacy. As Connelly and Clandinin (1995) recognize that beginning teachers are active agents (shaped by past and present experiences), pulling themselves into the future with their own inevitable social agendas (Rossi et al., 2015, p. 105), the researcher as a novice teacher in this study also transformed himself gradually to be assessment illiterate—assessment literate—assessment aliterate in a way of being safe in and adopting the teaching culture where he belongs, while questioning unchanged conventional assessment practices. Through the voice of a novice elementary teacher, this study provided the insiders’ perspectives and sociological understandings of elementary PE assessments, “explicitly linking the biographical to the institutional, the personal to the social” (R. Jones, 2009, p. 382). The novice teacher’s enactment of assessment literacy in elementary PE is not only related to themselves but also to the school culture, grade level team, and PE professionalism where he belonged. We, thus, should turn the academic focus more on the novice teacher’s assessment literacy from both sociological and individual perspectives in physical education and teacher education (PETE), and the autoethnography of novice teachers’ narratives on PE assessment practices to obtain further understanding of PE as well as the influencing factors of beginning teachers’ professional developments (Curry et al., 2008; Kelchtermans & Ballet, 2002a, 2002b; Schempp et al., 1993).
